# Auditory Hallucinations as Translational Psychiatry: Evidence from Magnetic Resonance Imaging

**DOI:** 10.4274/balkanmedj.2017.1226

**Published:** 2017-12-01

**Authors:** Kenneth Hugdahl

**Affiliations:** 1 Department of Biological and Medical Psychology, University of Bergen, Bergen, Norway; 2 Division of Psychiatry and Department of Radiology, Haukeland University Hospital, Bergen, Norway

**Keywords:** Auditory hallucinations, schizophrenia, functional magnetic resonance imaging, diffusion tensor imaging, neuroimaging, glutamate, dichotic listening

## Abstract

In this invited review article, I present a translational perspective and overview of our research on auditory hallucinations in schizophrenia at the University of Bergen, Norway, with a focus on the neuronal mechanisms underlying the phenomenology of experiencing “hearing voices”. An auditory verbal hallucination (i.e. hearing a voice) is defined as a sensory experience in the absence of a corresponding external sensory source that could explain the phenomenological experience. I suggest a general frame or scheme for the study of auditory verbal hallucinations, called Levels of Explanation. Using a Levels of Explanation approach, mental phenomena can be described and explained at different levels (cultural, clinical, cognitive, brain-imaging, cellular and molecular). Another way of saying this is that, to advance knowledge in a research field, it is not only necessary to replicate findings, but also to show how evidence obtained with one method, and at one level of explanation, converges with evidence obtained with another method at another level. To achieve breakthroughs in our understanding of auditory verbal hallucinations, we have to advance vertically through the various levels, rather than the more common approach of staying at our favourite level and advancing horizontally (e.g., more advanced techniques and data acquisition analyses). The horizontal expansion will, however, not advance a deeper understanding of how an auditory verbal hallucination spontaneously starts and stops. Finally, I present data from the clinical, cognitive, brain-imaging, and cellular levels, where data from one level validate and support data at another level, called converging of evidence. Using a translational approach, the current status of auditory verbal hallucinations is that they implicate speech perception areas in the left temporal lobe, impairing perception of and attention to external sounds. Preliminary results also show that amygdala is implicated in the emotional «colouring» of the voices and that excitatory neurotransmitters might be involved. What we do not know is why hallucinatory episodes occur spontaneously, why they fluctuate over time, and what makes them spontaneously stop. Moreover, is voice hearing a category or dimension in its own right, independent of diagnosis, and why is the auditory modality predominantly implicated in psychotic disorders, while the visual modality dominates in, for example, neurological diseases?

An auditory verbal hallucination (or simply auditory hallucinations, I will use the terms interchangeably), is typically defined as a sensory (perceptual) experience of hearing a voice in the absence of a corresponding external source (1). In the clinic, patients often report the subjective experience of someone speaking to them, often with negative comments about their behaviour and thoughts, how they dress, and what they say. The voices that patients experience hearing are, thus, typically emotionally negative, which differs from individuals hearing voices in the general population, who more often report positive comments and dialogues (2). Auditory hallucinations are one of the key symptoms of schizophrenia and are a major clinical characteristic of psychosis that severely handicaps the patients, who engage in recurrent conversations with the voices, which contributes to conceptual disorganization and impaired reality orientation (3). The content of auditory hallucinations, or what the voices actually say to the patients, varies from patient to patient, and although this has been the subject of numerous qualitative analyses, we still do not know if there is a core content of an auditory hallucination. Another clinical aspect of auditory hallucinations is capturing of attention, such that attention focus goes towards the inner voice rather than towards the outer surrounding environment. This has been the subject of psychological interventions (e.g., cognitive behaviour therapy), where the aim is to have the patient cognitively inhibit the inner voices and shift attention to the outer voices (4).

## Core dimensions

Hugdahl (5) listed three main cognitive characteristics of auditory hallucinations in that they have a perceptual dimension (“the voice speaks to the patient”), an attentional or executive dimension (“the patient cannot control the voice”), and an emotional dimension (“the voice is often evil”). In addition, the voices spontaneously fluctuate over time, which is often ignored in research, but might be a critical dimension when it comes to understanding the underlying neuronal and neurochemistry basis for auditory hallucinations (1). In this paper, I will review own research at the University of Bergen, along with some critical reflections on the status of current research, and what we actually know about the underlying mechanisms for this complex mental phenomenon.

## A symptom that crosses many borders

Although auditory hallucinations are primarily recognized as a schizophrenia symptom, this is a symptom of multiple psychiatric disorders, including bipolar disorder, post-traumatic stress disorder, depression, obsessive-compulsive disorder, personality disorders, and drug abuse. Auditory hallucinations are also symptomatic of psychiatric disorders and neurological diseases, such as Parkinson’s disease, epilepsy, dementia and Alzheimer’s disease. Finally, auditory hallucinations are found in both clinical and normal conditions; auditory hallucinations are also seen in the general population. Estimates of the prevalence of non-clinical auditory hallucinations vary between 5 and 20%, and a recent population study of the prevalence in Norway found that about 7.5% had experienced hearing voices (6). Although the voices heard by non-clinical individuals share characteristics with the voices heard by clinical individuals, there are some differences. Non-clinical voices are typically less viscous and intrusive, less cognitively controlling, and have an earlier debut (7,8). Taken together, these observations have led to the idea that auditory hallucinations might represent a mental phenomenon in its own right, independent of a clinical diagnosis, and when attached to a diagnosis, they are not uniquely linked to a psychotic disorder (9). This last point might have implications for a translational view of psychiatry since auditory hallucinations might better represent a state of mind than a unique sign of a psychotic breakdown. Thus, understanding the underlying mechanisms of auditory hallucinations might not only reveal something about a clinical symptom but also something of the core nature of how the mind works.

## Levels of Explanation (LoE) and translational psychiatry

**FIG. 1.**
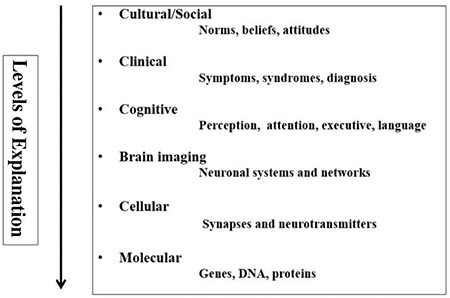
Graphical illustration of a Levels of Explanation approach for the understanding of auditory hallucinations.

Before turning to a review of some of the studies related to the underlying neuronal architecture behind auditory hallucinations, with a focus on the use of magnetic resonance imaging (MRI) for acquisition and analysis of the data related to the neurobiology of auditory hallucinations, I will first discuss what I (in other contexts) have labelled “Levels of Explanations (LoE)" (1,5). A LoE approach is illustrated in Figure 1 and shows how any mental phenomenon, including auditory hallucinations, can be approached from different LoE.

As outlined in Figure 1, auditory hallucinations are manifested at all levels, including cultural, clinical, cognitive, brain-imaging, cellular-transmitter, and molecular levels, and a complete understanding of this complex mental phenomenon would require a comprehensive theory or model that would encompass all levels, moving vertically. This is, however, a paramount task, which is beyond the expertise of a single researcher, and would require extensive collaboration in a translational perspective. What is required, however, is that we extend the current trend in research of expanding horizontally, where researchers remain at their favourite level and acquire ever more data using more advanced methods and analyses. To expand our knowledge of the underlying neuronal mechanisms for auditory hallucinations we, therefore, need to move vertically, at least to be able to go from one level to the next, even though it would be premature to claim that we have data for a comprehensive theory of auditory hallucinations that encompass all six levels. In the following, I will illustrate this by presenting data that allow us to piece together how the phenomenology of “hearing a voice” at the clinical level have unique correlates at the cognitive, brain and cellular levels (1). I will start by reviewing data from functional neuroimaging, using functional magnetic resonance imaging (fMRI).

## Functional neuroimaging

I will pursue a view of auditory hallucinations as essentially perceptual phenomena, and that a speech perception model has a better fit with the clinical level than a speech production model. As pointed out by (10), the behavioural and neuroimaging evidence for speech production models, such as inner speech, is limited and inconclusive. Also, most voices are perceived in third-person, which is at odds with an inner speech view, which would predict that the individual experiences his or her own voice, at least occasionally, which is not the case.

## The “classic” fMRI finding

**FIG. 2.**
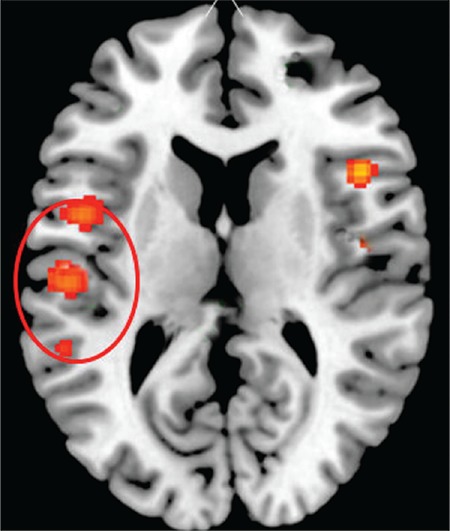
fMRI activations in the speech areas in the upper posterior part of the temporal lobe during auditory hallucinations (adapted from reference 11).

At our laboratory in Bergen, Kompus et al. (11) conducted a meta-analysis of existing fMRI studies on the localization of activation patterns when patients reported having hallucinatory experiences while in the scanner. The core finding was of spontaneous activation of an auditory network, with the centre of gravity in the left superior temporal gyrus, extending into the superior temporal sulcus, and covering the classic speech perception areas. The findings of Kompus et al. (11) perfectly overlap with fMRI speech perception studies in healthy individuals. Figure 2 shows how the auditory and speech perception network is activated in healthy individuals when listening to simple speech sounds, such as consonant-vowel syllables (12). Comparing the activation pattern in the healthy individual (left-hand panel of Figure 2) with the corresponding activation pattern reported by Kompus et al. (11) (right-hand panel of Figure 2) shows a clear overlap of the patterns. This validates that the activation pattern seen during spontaneous auditory hallucinations in the absence of an external speech sound source is almost identical to the activation pattern in non-hallucinating individuals in the presence of an external speech sound source.

To add to this, similar patterns were also found in another meta-analysis published about the same time by Jardri et al. (13) France, although they also found spontaneous activation in the prefrontal cortex, especially on the right side. This observation fits nicely with the findings of Sommer et al. (14) at the Utrecht University Medical Center, Netherlands; that activation of speech production areas in the right hemisphere might contribute to the single word or low complexity sentences that often characterizes auditory hallucinations, and which the right hemisphere is capable of. A third meta-analysis (15) found that while state-effects involved the frontal cortex, trait-effects primarily involved the auditory cortex, an interpretation that would also fit the available data from other studies.

From the “classic” studies by (11,13,15), which were mainly concerned with single area activations, recent fMRI studies focus more on activation of cortical networks and network connectivity (16,17,18,19,20). Here the term trait-effects indicates that patients have a tendency to experience auditory hallucinations but might not be experiencing them at the time of examination (which would be called a state-effect). These studies have, however, been inconclusive in the sense that, although most studies point to a fronto-temporo-parietal network that is activated in auditory hallucinations, the results regarding the direction and strength of the activation are inconclusive; some studies show increased cortical connectivity, while others show decreased connectivity (21,22).

## The “paradoxical” fMRI finding

An interesting question that emerged after the initial fMRI studies of spontaneously driven activations during hallucinatory experiences was what would the activation pattern look like if these patients, in addition to their “inner voices”, were also presented with “outer voices” (real speech sounds through headphones) while in the scanner. I had originally predicted that the activation caused by the outer voices would add to the activation caused by the inner voices, such that the net sum would be an increase in strength and or extension. However, the finding in the Kompus et al. (11) study was, to our surprise, the opposite; the two sources seemed to compete for processing resources, such that the net sum was of a subtraction and the inner voices seemed to inhibit processing of the outer voices. This could either be a blocking of the external source, in which case the perceptual system would be down-regulated or shut-down, or an attentional effect, such that the patient is unable to attend to the outer voices, in which case the cognitive system would be down-regulated or shut down. We do not know which of these hypotheses is true and, in addition, there could be a third explanation in which the auditory signal never reaches the processing areas in the temporal lobe, in which case there would be a signal-conduction failure in the basal ganglia.

It could be argued that this paradoxical finding is due to a methodological limitation with fMRI, such that the inner voices recruit all the available oxygen, with the result that there is a ceiling effect with no further oxygen available for the outer voices. This would have been a feasible explanation had it not been that similar paradoxical effects have also been reported for positron emission tomography (23) and electroencephalography/event-related potential (24), which are methods that do not depend on oxygen consumption.

To summarize this section, it is probably not an exaggeration to say that the upper posterior part of the left temporal lobe and the secondary auditory cortex is implicated in auditory hallucinations. This was also demonstrated in a recent study by Moseley et al. (25), where they used a transcranial magnetic stimulation technique over the left temporal cortex to induce hallucination-like experiences in individuals prone for such experiences. When it comes to connectivity studies, the situation is more complex and unsettled, with studies showing both increased and decreased connectivity. Finally, with regard to the paradoxical finding of competition between inner and outer voices, I think that this was nicely summarized by (21) in their recent review of imaging studies of auditory hallucinations. These authors wrote that: *“The observation that auditory hallucinations reduce responsivity to external speech within STG and related regions such as MTG has led to the ‘saturation theory’ whereby the signal driving hallucinations competes for common neurophysiological resources with those used for perceiving external speech”* (p. 15).

## Structural imaging and white matter connectivity

**FIG. 3.**
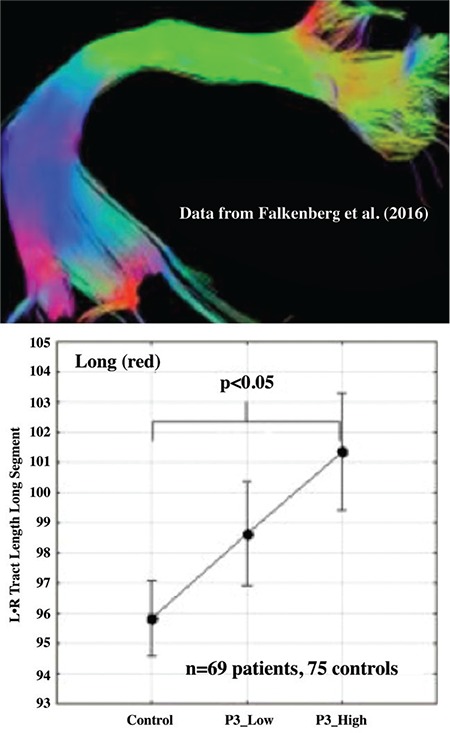
Analysis of fibre-tract length (red fibres) in hallucinating (P3_high, n=31) and non-hallucinating patients (P3_low, n=38) compared to healthy controls (n=75) for sections of the arcuate fasciculus (DTI data, see text for further details). Results adapted from reference (26), segmentation of the arcuate fasciculus from reference (27).

Returning to a LoE perspective, functional connectivity studies would be strengthened if it could be shown that, for example, white matter connection between temporal and frontal areas and, in particular, between speech perception areas in the temporal lobe and speech production areas in the frontal lobe, could be shown to have stronger or weaker white matter connections in frequent hallucinators. This would then be an example of what I have called "converging evidence", i.e. when data obtained with one method are validated by data obtained with another method. The most important fibre tract connecting the temporal and frontal language areas is the arcuate fasciculus, which extends from the posterior temporal lobe to the inferior frontal region in the lateral plane. White matter connections can be measured and visualized with diffusion tensor imaging (DTI) and, in a recent study from our laboratory (26), we found that schizophrenia patients with frequent and severe hallucinations had larger fibres in the long segments of the arcuate fasciculus white matter fibre tract compared to healthy controls. Figure 3 shows the white matter segments of the arcuate fasciculus, colour-coded according to the sectioning and segmentation suggested by (27). Hallucinations were quantified from the question in the Positive and Negative Syndrome Scale (PANSS) interview questionnaire related to hallucinations (P3), and split for frequent (P3_high) and infrequent (P3_low) for a score of “4” on the PANSS P3-item.

The graph in Figure 3 shows that the tracts for the long fibres (red colour in the segmented illustration) connecting the posterior and anterior sections of the arcuate fasciculus bundle were significantly longer in the frequent hallucinating group compared to healthy controls, while there was no significant difference for patients with less frequent and severe hallucinations. Using DTI data, Falkenberg et al. (26) found that functional connectivity was stronger in hallucinating patients and, thus, that the signal-flow between the speech perception and production regions could have multiple destinations and origins, which could result in the subjective experience of hearing a voice in the absence of an external sound source.

## Cognitive level - Convergence of evidence

If auditory hallucinations block activation to external speech sounds at the brain-level, such experiences should then also interfere with the subjective experience (perception) of speech sounds (i.e., must show that the effect also exists at the cognitive level). Another way of expressing this is to say that, for a finding to be valid, there should be converging evidence from multiple levels and methods. Convergence of evidence could also be obtained at the same level, but with different methods, like what was shown above when DTI data converges with fMRI data.

**FIG. 4.**
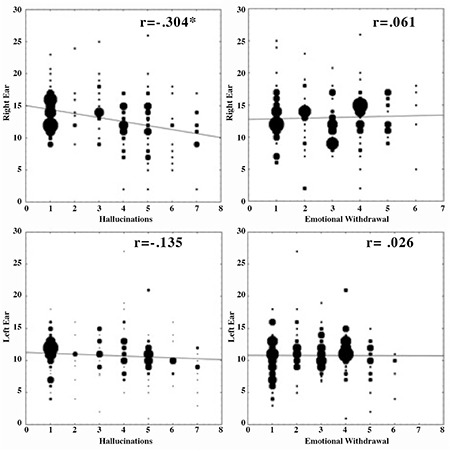
Scatter-plots with correlations for performance on the dichotic listening task and frequency and severity of auditory hallucinations quantified from the PANSS P3 item. Data from reference (28). Asterisk indicates statistical significance, p<0.05

Hugdahl et al. (28) used a dichotic listening approach, which is a behavioural, cognitive method, to localize the speech perception centre in the brain. This method uses the simultaneous presentation of two different simple speech sounds, typically consonant-vowel syllables (e.g., /ba/, /pa/, /ga/); one in the right and the other in the left ear (29,30). Since the right-ear syllable in a dichotic situation has direct input to the processing centre in the left temporal lobe, while the left ear stimulus has to be transferred over the corpus callosum, most people report more correct syllables from the right ear; something called a right-ear advantage (31,32). Having the classic fMRI finding as a back-drop, in the Hugdahl et al. (28), we predicted that the number of correctly reported syllables from the right ear would decrease as a function of the increase in frequency and severity of auditory hallucinations (we predicted a negative correlation between right ear performance and hallucinations). Hallucinations were quantified from responses to the PANSS (33) interview questionnaire (question P3, related to hallucinations). The results are shown in the left-hand panels of Figure 4 and revealed a significant negative correlation between the right ear performance, while the corresponding correlation for the left ear performance was non-significant. An interesting aspect of the left-hand panel data in Figure 4 is that the data were based on a relatively large sample of 160 patients with a diagnosis of schizophrenia. The patients came from Norway, Turkey and the United States, and differences in culture (particularly religion) were partially controlled for took, which would have relevance for a cultural level explanation (34).

Perhaps of greater interest, when dichotic listening performance was correlated with a negative symptom from the PANSS interview questionnaire, correlations were all non-significant and close to zero (see right-hand panels of Figure 4). Such a pattern would be expected since a negative symptom (in this case “emotional withdrawal”, N2) should not be related to the functional integrity of the speech perception areas in the posterior temporal lobe. In conclusion, the findings with the dichotic listening technique, at the cognitive behavioral level, validate and support the findings at the brain-imaging level, such that when activation is blocked or suppressed in hallucinating patients when exposed to an external speech sound, this also has direct consequences for the perception and processing of an external sound stimulus, which would be predicted from a perceptual model of auditory hallucinations. From a clinical point of view, it could be argued that reduced perceptual accuracy of external auditory events is not directly observable in the behaviour of patients. However, looking into the experimental literature reveals that hallucinating patients or hallucinating-prone individuals have problems with pitch perception deficits for basic auditory stimuli (26), are impaired in recognition of familiar voices (36) and recall of previously presented voices (37), and have impaired ability to analyze speaker identity (38). These patients are also impaired in voice identity recognition (39), and perform worse than non-hallucinators and controls for pitch discrimination of unmodulated tones and auditory streaming (40). Thus, it is clear that auditory hallucinations impair or reduce perceptual accuracy for auditory events and stimuli across environmental conditions.

## What may be causing the changes in activation, the neurochemistry of auditory hallucinations

**FIG. 5.**
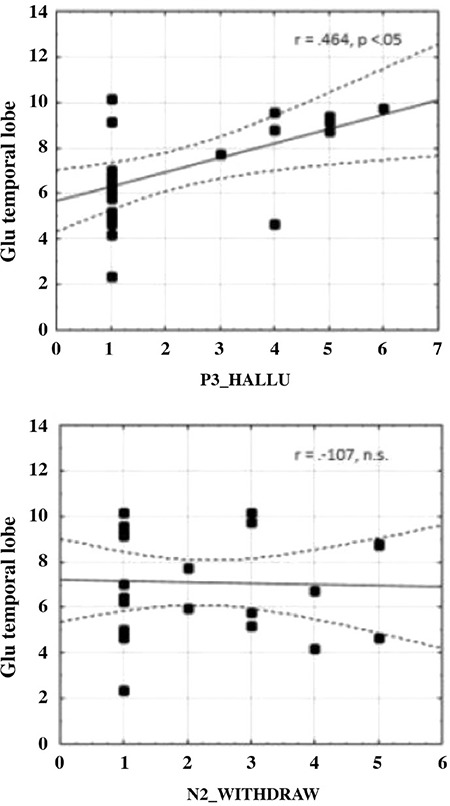
Scatter-plots with correlations for glutamate + glutamine (Glx) levels and frequency and severity of auditory hallucinations. Data from reference (41).

A critical question seldom asked in hallucinations research, or in functional brain-imaging research in general, is what is causing the change in neuronal firing and subsequent increase in hemodynamic metabolism and oxygen inflow. However, every regional change in the blood-oxygenation level dependent response as a consequence of an internal or external stimulus will have a corresponding change at the cellular level, meaning a change in receptor and transmitter function and activity. This has traditionally been an obstacle since it has been problematic to measure changes in transmitter function in vivo in humans. The introduction of magnetic resonance spectroscopy (MRS) and the implementation of MRS sequences on standard MR machines by most major MR vendors has now made this possible. MRS allows for the quantification of the minute concentration of certain brain metabolites, which also acts as transmitters, in particular, the excitatory transmitters glutamate and glutamine (measured as the sum, Glx), and the inhibitory transmitter gamma-amino-butyric acid (GABA). In the first study of the neurochemistry of auditory hallucinations, Hugdahl et al. (41) reasoned that since an auditory hallucination is a positive symptom, and as such an excitatory phenomenon, it would not be unreasonable to hypothesize that an excitatory transmitter may be involved in the change in activation observed at the brain-imaging level. This was tested empirically in a group of schizophrenia patients and healthy control subjects, where the schizophrenia group was further split into frequent and non-frequent hallucinating subgroups. In addition to having been through an fMRI session, the subjects also went through an MRS sequence of about 5 min, where glutamate and a range of other metabolites were measured from temporal and frontal lobe areas. Technical limitations make it impossible to acquire metabolite concentrations from all regions in the brain at the same time, so one typically uses a few pre-selected areas, called MRS voxels, of about 8-27 mL volume. The MRS sequence is typically run in a resting-state condition, although it is possible to acquire metabolite information also in a task-situation, in which case the session is called functional MRS, analogous to functional MR. Hugdahl et al. (41) found that schizophrenia patients had lower Glx concentrations in both temporal and frontal lobe areas (where the MRS voxels were placed) than controls. However, when we analyzed the two sub-groups of schizophrenia patients separately, it turned out that the effect of lower Glx levels in the schizophrenia group was entirely driven by the non-frequent hallucinating group, while the frequent hallucinators had significantly higher levels. We further correlated the Glx levels with frequency and severity of auditory hallucinations, as taken from the PANSS P3 item, described above. The results are shown in Figure 5, with significant positive correlations for both the temporal and frontal voxels, and (as for the dichotic listening data) we also correlated Glx levels with the same negative symptom, with insignificant findings.

From the results in Figure 5, it seems as if auditory hallucinations and corresponding receptor functioning are coupled such that an increase in glutamate in the synaptic cleft might underlie the call for redistribution of blood flow to the same region. This year, a second Glx study appeared which essentially replicated the increase in Glutamate found by Hugdahl et al. (41), first-authored by Branislava Curcic-Blake and originating from the Groningen University Medical Center in the Netherlands (42). Moreover, the Ćurčić-Blake et al. (42) study had a subgroup of life-time hallucinators that was compared with a subgroup of patients that had never hallucinated, extending the data basis. The authors concluded that: *“The finding that Glx levels appear to be higher in patients with lifetime auditory hallucinations than in patients without lifetime auditory hallucinations, is in line with the only previous study that related Glx to auditory hallucinations (Hugdahl et al., 2015), and supports the idea that glutamatergic metabolites are a mediating factor in auditory hallucinations.”* (p. 8). However, the Ćurčić-Blake et al. (42) study did not report on a significant correlation between PANSS P3 scores and Glx levels, which was reported by (41). This could be due to methodological differences between the two studies, Ćurčić-Blake et al. (42) only acquired data from the prefrontal cortex, or from differences in patient selection. Nevertheless, there seems to be evidence for a connection between Glx and Glutamate on the one hand and increased frequency and severity of auditory hallucinations on the other, a finding that could lead to the development of new therapeutic interventions. A second conclusion is that these findings nicely fit into a LoE model, where findings at the cellular level support and validate findings at the brain-imaging, cognitive and clinical levels.

## The emotional valence of auditory hallucinations

**FIG. 6.**
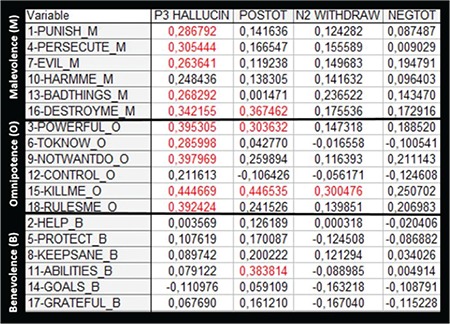
Correlation coefficients for correlations (red = significant correlations) between scores on the PANSS P3 and N2 items and total score for positive (POSTOT) and negative (NEGTOT) symptoms, with scores on the BAVQ-R self-report scale. See text for further details.

A final issue to be reviewed in this article is the remarkable fact that up to 80% of hallucination schizophrenia patients experience that the voices are vicious and evil and carry a negative emotional tone or valence. This is probably a major clinical feature of auditory hallucinations, which also creates anticipatory anxiety and frustration “when will the voices next appear?”. If the voices were positive, there would not be the need to allocate cognitive effort to avoid and get away from the voices as when they are negative with malevolent comments and sometimes even commands to perform acts the patients do not want to commit. This is a puzzling observation since there is no a priori reason why the voices that patients experience should have a predominantly negative emotional valence. Our laboratory has been generating data on the emotional valence of auditory hallucinations and investigating whether increasing negative valence goes together with increasing frequency and severity of auditory hallucinations. For this purpose, we have used a self-report questionnaire with questions about the content of the voices [the Beliefs About Voices Questionnaire (BAVQ)] originally developed by Chadwick and Birchwood (43,44) in the UK. The BAVQ questionnaire contains statements regarding the content and intention of the voices, like “My voice is evil”, “My voice is very powerful”, or “My voice wants to help me”, to which the patient makes a mark on a 4-scale response sheet ranging from disagreeing to agree strongly. Some of the statements have a malevolent content (they are negative in nature), some are benevolent (they are positive in nature), and some have an omnipotent content (they have power over the patients, which also in all essence a malevolent statement). We have recently analyzed BAVQ-data from 160 patients, where we correlated the BAVQ scores for each of the 18 statements (about malevolent, benevolent and omnipotent voice content) with the PANSS P3 (hallucinations), total positive symptoms score, N2 (emotional withdrawal), and total negative symptoms score, with the hypothesis that the malevolent statements would correlate positively with PANSS P3 scores, but not with PANSS N2 scores. The results are seen in Figure 6, where the BAVQ statements have been re-arranged into malevolent (M), benevolent (B), and omnipotent (O) items along the vertical axis, with the PANSS items along the horizontal axis. The scores for the PANSS and BAVQ items were correlated with Spearman’s rank-order correlation (significance set at p<0.05) because of the restricted range of the scores for both the PANSS and BAVQ questionnaires.

As can be seen in Figure 6, there were significant correlations between BAVQ M- and O-statements and PANSS P3 items, but no significant correlations for BAVQ B-statements. Also, there were a few significant correlations for the sum of PANSS positive symptoms, not seen in Figure 6, which could indicate that other positive symptoms (e.g., P1 “Delusions”) also correlate with negative content. Finally, there were one or two significant other correlations, that probably represent random effects due to the relatively large matrix size. Taken together, these correlations show that increasing frequency and severity of auditory hallucinations in a patient group with schizophrenia goes together with increasing negative content, as seen in the significant positive correlations between the M- and O-statements and the absence of significance for the B-statements.

## The “VOICE” - model

**FIG. 7.**
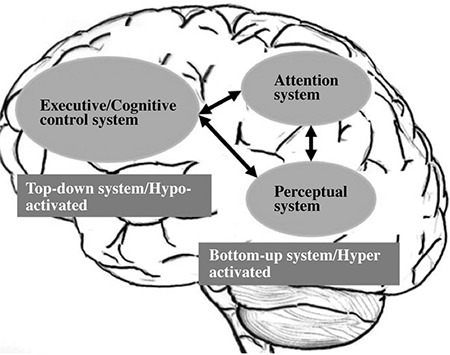
The VOICE-model for auditory hallucinations, see text for further details. Adapted from Hugdahl, 2009.

Figure 7 shows an illustration of a neurocognitive model to understand the neuronal mechanisms involved in auditory hallucinations.

The model assumes two major functional systems at the cognitive and brain levels; a bottom-up system localized to an auditory-perceptual network in the temporal lobe, and a top-down system localized to an attention-executive network localized to frontal lobe areas (45). An auditory hallucination is then thought to be the result of hyper-excitation of the bottom-up system, such that neurons in this region spontaneously fire in the absence of a triggering external stimulus, and hypo-excitation of the top-down system, such that attention is not regulated appropriately to an external event and internal events are not correspondingly inhibited or suppressed. The VOICE-model was originally presented in (45), but has been revised to accommodate recent fMRI connectivity studies that point to the importance of incorporating how these systems or large-scale networks interact, something that can be labelled network inter-connectivity in contrast to the more common analysis of network intra-connectivity (i.e., how nodes within a network interact). However, as mentioned above, it is at present unclear how the networks interact, and if there is increased or decreased connectivity strength in hallucinating patients.

## The role of the amygdala

At the brain-level explanation, a key question about the emotional colouring of auditory hallucinations is: what brain structures are involved? From knowledge of the role played by the amygdala and the limbic system in emotional fear responses (46), it could be hypothesized that the amygdala is also involved in the emotional colouring of auditory hallucinations. Unpublished data from our laboratory show that the thalamus-amygdala pathways are more activated in hallucinating patients. This could indicate that auditory hallucinations activate the so-called “low route”, which bypasses the cortex and initiates an emotional response non-consciously. These data are preliminary and from a small sample but may, nevertheless, be of value by identifying new neuronal pathways in the brain for the expression of auditory hallucinations.

In this review paper, I have presented results from our laboratory at the University of Bergen, Norway that has hopefully illustrated the LoE approach to the understanding of the mental phenomenon in general, and auditory hallucinations in particular. More precisely, I have tried to illustrate a LoE approach for auditory hallucinations by showing how data acquired at the brain-imaging level are validated and supported by corresponding data acquired at higher (cognitive) lower (cellular) levels, where transmitter and metabolite concentrations are recorded from selected brain regions and correlated with clinical data. A LoE approach is also relevant from a translational perspective since it brings together laboratory and clinical data, which is the classic definition of a translation perspective on health and disease. I have also presented a theoretical model for capturing some of the core neurocognitive characteristics of auditory hallucinations, with bottom-up versus top-down identified as a key concept. In this context, bottom-up processing refers to spontaneous neuronal firing in the speech areas in the temporal lobe, which gives rise to a perceptual experience at a phenomenological level of “hearing someone talking to me”. The top-down processing is the conscious act of, for example, shifting attention from one source of input to another (e.g., when shifting attention from the inner to the outer voices, and to be able to suppress or ignore the inner voices). We believe that a critical aspect of auditory hallucinations is an imbalance in how the bottom-up and top-down systems interact, such that the inner voices are not suppressed and attention not shifted away in hallucinating patients. This is often the target of various cognitive behaviour therapy approaches aimed at teaching patients to cognitively cope with their voices. It could be speculated whether the fluctuations were seen in almost all patients with regard to how the voices come-and-go over the course of a day, although with great variability between patients, may reflect a fundamental imbalance between excitatory, such as glutamate, and inhibitory, such as GABA, neurotransmitters. An often ignored clinical fact is that auditory hallucinations are seldom constantly present, although again with great variability, and a question that arises is whether the spontaneous cessation of an auditory hallucinations means that the Glutamate-GABA balance is temporarily restored, and then it goes out of phase or balance when an auditory hallucinations episode is about to break out again the next time. There are some data to support this hypothesis; many hallucinating individuals do not report a specific triggering event for the onset of a hallucinatory episode (47). Instead, these episodes seem to occur spontaneously (“out of the blue”), which could mean that the onset and offset of an episode is driven more by transmitter imbalance at the cellular and neurochemistry level than by external events, or by cognitive distortions that are secondary to the initiation event. Using a translational approach, the current view of auditory verbal hallucinations is that they implicate speech perception areas in the left temporal lobe, thereby impairing the perception of and attention to external sounds. Preliminary results show that amygdala is implicated in the emotional «colouring» of the voices and that excitatory neurotransmitters might be involved. Outstanding questions include, why hallucinatory episodes occur spontaneously, why they fluctuate over time, and what makes them spontaneously stop? Moreover, is voice hearing a category or dimension in its own right, independent of diagnosis, and why is the auditory modality predominantly implicated in psychotic disorders, while the visual modality dominates in, for example, neurological diseases?
